# In Situ Reduction-Generated Ag^0^ Plasmonic Sites on Ti_3_C_2_/Ag_2_NCN Schottky Heterojunctions for Efficient Photocatalytic Tetracycline Degradation

**DOI:** 10.3390/molecules31172955

**Published:** 2026-08-23

**Authors:** Haidong Yu, Hua Deng, Jincheng Wang, Xiaohe Sun, Jingyu Liu, Ping Qu, Jie Wu

**Affiliations:** 1 Langfang Natural Resources Comprehensive Survey Center, China Geological Survey, Langfang 065000, China; yuhaidong1992@163.com (H.Y.); denghua137@126.com (H.D.); 18129361330@163.com (J.W.); 18953588146@163.com (X.S.); 17692600460@163.com (J.L.); 2 Center for Geophysical Survey, China Geological Survey, Langfang 065000, China

**Keywords:** Ti_3_C_2_ MXene, Ag_2_NCN, Schottky heterojunction, photocatalytic degradation, tetracycline

## Abstract

Constructing Schottky heterojunctions with plasmonic components offers a promising route to enhance photocatalytic performance, yet the synergistic roles of the Schottky barrier and localized surface plasmon resonance (LSPR) in pollutant degradation remain insufficiently elucidated. Herein, a series of Ti_3_C_2_/Ag-Ag_2_NCN (TAN) composites with varied Ag loadings was prepared via an *in situ* precipitation–chemical reduction method. The pseudo-first-order rate constant of the TAN-30 heterojunction reached roughly 7.0 times the value of bare Ag_2_NCN, while its tetracycline degradation efficiency under visible light reached 87.0% at 240 min. Moreover, the heterojunction exhibited outstanding reusability over five successive runs. Comprehensive characterizations reveal that the Schottky barrier at the Ti_3_C_2_/Ag_2_NCN interface effectively suppresses photogenerated carrier recombination, while the LSPR effect of metallic Ag^0^ broadens the light absorption range and elevates the local surface temperature, synergistically accelerating charge migration. The dominance of h^+^ and •O_2_^−^ among the reactive species was established by both radical trapping assays and ESR spectroscopic analysis. This work provides mechanistic insights into LSPR-enhanced Schottky heterojunctions and offers a rational design strategy for MXene-based photocatalysts toward efficient antibiotic wastewater treatment.

## 1. Introduction

The increasing consumption of fossil fuels and the concomitant environmental pollution have spurred intense research into sustainable and clean energy technologies [1,2,3]. Tetracycline (TC) is a globally prevalent antibiotic contaminant in water bodies, arising from pharmaceutical and agricultural sources [4]. Its persistence, ecotoxicity, and role in propagating antibiotic resistance genes make it recalcitrant to conventional treatment. Photocatalysis offers a promising solution for TC degradation. Solar-driven photocatalysis has emerged as a promising approach for both energy conversion and environmental remediation, as it enables the direct utilization of abundant and renewable solar energy [5,6]. Among various photocatalytic applications, the degradation of persistent organic pollutants in wastewater, particularly antibiotics such as tetracycline (TC), has attracted considerable attention due to the growing threat of antibiotic resistance and the inadequacy of conventional treatment methods [7,8].

In recent years, silver cyanamide (Ag_2_NCN) has attracted growing interest as a novel semiconductor photocatalyst with a narrow band gap (~2.5 eV) and favorable band edge positions for visible-light-driven redox reactions [9,10]. Its unique [NCN]^2−^ anion structure facilitates efficient charge separation, yet its photocatalytic performance remains constrained by moderate carrier mobility and insufficient surface reactivity [11,12]. Coupling Ag_2_NCN with a highly conductive support to form a Schottky heterojunction represents a rational strategy to address these limitations, as the built-in electric field at the interface can effectively drive photogenerated electrons toward the conductive component, thus suppressing bulk recombination [13].

Two-dimensional transition metal carbides and nitrides, namely MXenes, have recently emerged as promising cocatalyst materials owing to their metallic conductivity, tunable surface chemistry, and large specific surface area [14,15]. Ti_3_C_2_T_x_, the most widely studied MXene, possesses excellent electrical conductivity and abundant terminal groups (−OH, −F, =O) that facilitate the anchoring of nanoparticles and the formation of intimate interfacial contacts [16,17,18]. When coupled with semiconductors, Ti_3_C_2_T_x_ can function as an efficient electron sink, promoting charge separation through the Schottky barrier [19,20,21]. However, simply integrating Ti_3_C_2_T_x_ with Ag_2_NCN may not fully exploit the potential of the composite system, as the plasmonic properties of metallic Ag nanoparticles could introduce additional light-harvesting capabilities that further enhance photocatalytic performance [22]. 

Metallic Ag nanoparticles exhibit localized surface plasmon resonance (LSPR) effects, which can significantly extend the light absorption range of composite photocatalysts into the visible and near-infrared regions [23]. More importantly, LSPR-generated hot electrons can be injected into the adjacent semiconductor, while the localized photothermal effect elevates the reaction temperature and accelerates carrier migration [24]. In this context, the *in situ* formation of Ag^0^ on the Ag_2_NCN surface during composite synthesis offers a distinct advantage: the intimate interface between Ag^0^ and Ag_2_NCN, combined with the subsequent coupling with Ti_3_C_2_T_x_, could establish a ternary synergistic system where the Schottky barrier and LSPR effect jointly promote charge separation and light absorption. Nevertheless, the interplay between these mechanisms in the Ti_3_C_2_/Ag-Ag_2_NCN system remains poorly understood, and the optimal Ag loading for achieving synergistic enhancement has yet to be systematically investigated.

Herein, we report the design and fabrication of a Ti_3_C_2_/Ag-Ag_2_NCN (TAN) Schottky heterojunction via an *in situ* precipitation–chemical reduction route, in which Ag^0^ is generated concurrently with Ag_2_NCN deposition onto Ti_3_C_2_T_x_ nanosheets. Ag loading was systematically varied to optimize photocatalytic activity toward tetracycline degradation under visible light irradiation. Through a combination of structural characterization, photoelectrochemical measurements, and radical trapping experiments, we elucidate the synergistic roles of the Schottky barrier and the LSPR effect of *in situ* formed Ag^0^ in enhancing charge separation and reactive oxygen species (ROS) generation. This work provides mechanistic insights into MXene-based plasmonic heterojunctions and offers a rational design strategy for efficient antibiotic wastewater treatment.

## 2. Results and Discussion

### 2.1. Structural Characterization

The synthesis begins with the selective etching of aluminum layers from the bulk Ti_3_AlC_2_ MAX precursor using hydrofluoric acid, resulting in a multilayered Ti_3_C_2_ product with an accordion-like morphology [25]. Following this, intercalation with dimethyl sulfoxide (DMSO) combined with ultrasonication effectively exfoliates the stacked layers into few-layered nanosheets [26]. Upon subsequent AgNO_3_ addition, the negatively charged Ti_3_C_2_ surface readily captures Ag^+^ cations via electrostatic interactions, owing to its zeta potential, which arises from abundant terminal functional groups [27]. The introduction of H_2_NCN then provides NCN^2−^ species that react with these adsorbed Ag^+^ ions to nucleate Ag_2_NCN nanoparticles, which are tightly anchored onto the Ti_3_C_2_ support. Concurrently, the strong reducing capability of the low-valence titanium species existing within the Ti_3_C_2_ lattice induces the partial reduction of Ag^+^ to elemental Ag^0^ [28]. 

**Figure 1:**
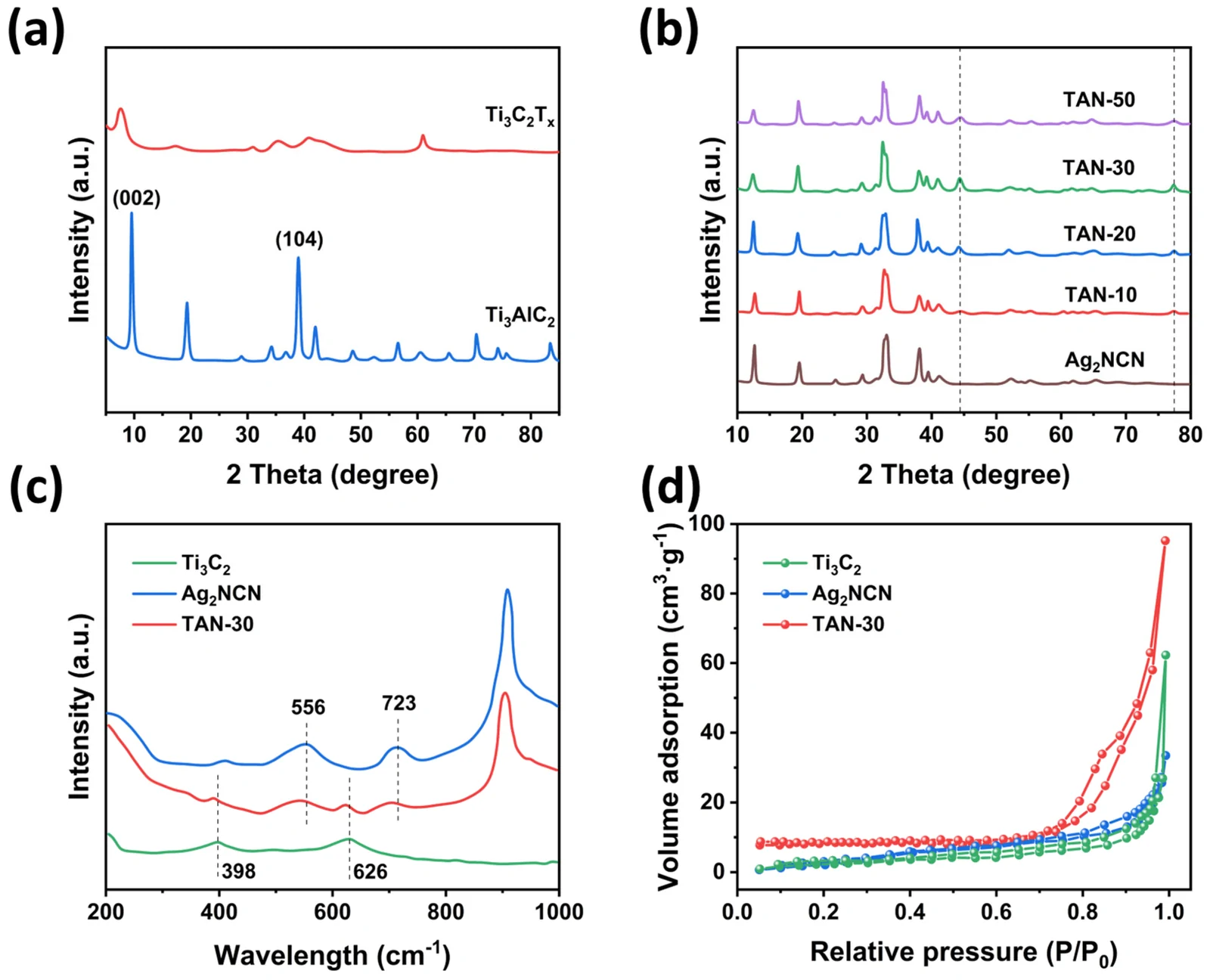
(**a**) XRD patterns of precursor Ti_3_AlC_2_ and product Ti_3_C_2_; (**b**) XRD patterns of Ag_2_NCN and its hybrids with varying Ti_3_C_2_ contents; (**c**) Raman spectra of Ti_3_C_2_, Ag_2_NCN, and TAN-30 for comparison; (**d**) N_2_ adsorption-desorption isotherms of Ti_3_C_2_, Ag_2_NCN, and TAN-30.

The crystal phase composition of the as-prepared samples was examined by X-Ray diffraction. In Figure 1a, the pristine MAX phase displays sharp reflections that match well with the reported Ti_3_AlC_2_ pattern [29]. Following HF etching and DMSO-assisted sonication exfoliation, the considerable weakening of the diffraction peaks, the near disappearance of the (104) reflection at approximately 39°, and the downshift in the (002) peak to a lower Bragg angle collectively confirm the successful removal of the Al layers and the conversion of the MAX precursor into Ti_3_C_2_ nanosheets [16]. Figure 1b presents the XRD profiles of Ag_2_NCN and the Ti_3_C_2_/Ag-Ag_2_NCN hybrids with varying Ti_3_C_2_ loadings. All samples show well-defined peaks attributable to Ag_2_NCN, indicating that the incorporation of Ti_3_C_2_ does not alter the lattice structure of Ag_2_NCN. The low Ti_3_C_2_ loading and its uniform distribution in the hybrids account for the absence of any detectable Ti_3_C_2_ reflections. Additionally, the hybrid XRD patterns reveal extra peaks corresponding to metallic Ag, which arise from the partial reduction of Ag^+^ by the low-valence Ti species present in Ti_3_C_2_ [21]. The emergence of Ag (200) and (311) reflections at 44.15° and 77.56° with plateaued intensities, observable when the Ti_3_C_2_ suspension addition reaches 30 mL (20 mg/mL), signals the establishment of a stable hybrid system.

The Raman spectra of Ti_3_C_2_, Ag_2_NCN, and the TAN-30 hybrid are presented in Figure 1c. Pristine Ag_2_NCN shows distinct bands at 556 and 723 cm^−1^, whereas Ti_3_C_2_ has peaks at 398 and 626 cm^−1^. For TAN-30, these characteristic signals are weakened and shifted to lower wavenumbers relative to the pristine components, indicating the coexistence of Ti_3_C_2_ and Ag_2_NCN in the composite as well as a notable interfacial interaction between them. BET specific surface areas were derived from N_2_ adsorption–desorption isotherms (Figure 1d). All samples exhibit type IV isotherms with H3 hysteresis loops, characteristic of mesoporous structures. The calculated surface areas for Ti_3_C_2_, Ag_2_NCN and TAN-30 are 12, 9 and 20 m^2^ g^−1^, respectively. The hybrid TAN-30 shows a larger surface area than either pristine component, owing to the suppressed self-aggregation upon combining Ag_2_NCN with Ti_3_C_2_ [30]. This enlarged area affords additional active sites and facilitates ion transport, thereby promoting photocatalytic activity.

**Figure 2:**
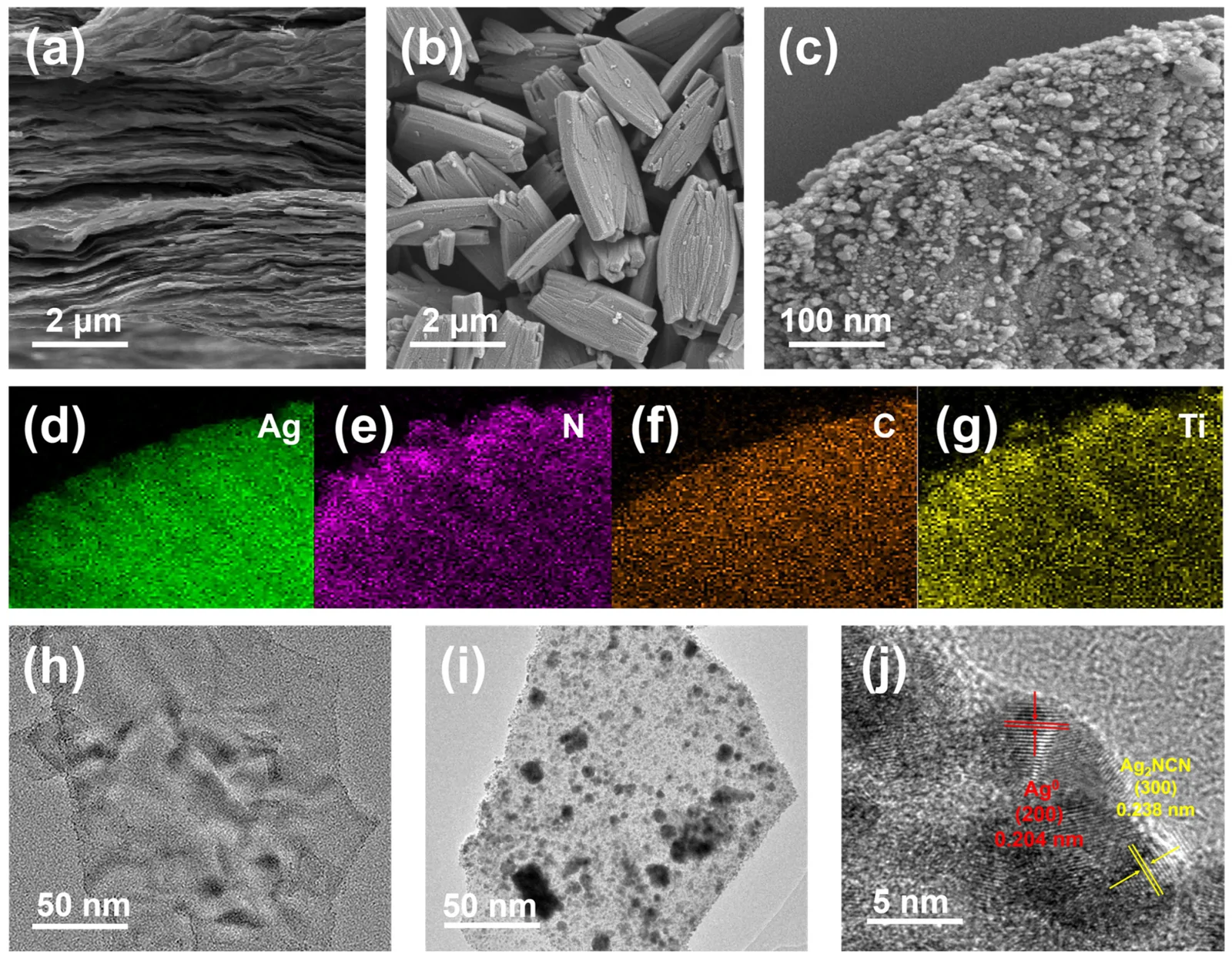
SEM images of (**a**) Ti_3_C_2_, (**b**) Ag_2_NCN, and (**c**) TAN-30 and (**d**–**g**) elemental mapping for TAN-30, TEM images of (**h**) Ti_3_C_2_ and (**i**) TAN-30 and (**j**) HRTEM images of TAN-30.

The surface morphologies of Ti_3_C_2_, Ag_2_NCN, and TAN-30 were examined via SEM, with the corresponding micrographs presented in Figure 2. The Ti_3_C_2_ sample exhibits a typical accordion-like multilayered structure with a smooth surface, confirming the complete removal of Al layers from the bulk Ti_3_AlC_2_ precursor after HF etching. Following DMSO intercalation and ultrasonic exfoliation, few-layer Ti_3_C_2_ nanosheets featuring distinct two-dimensional layered configurations were clearly observed (Figure 2a). In contrast, Ag_2_NCN displayed a micrometer-scale solid architecture composed of rectangular plate-like particles (Figure 2b). For TAN-30, which was prepared with the addition of 30 mL (25 mg·mL^−1^) Ti_3_C_2_ suspension, Ag/Ag_2_NCN nanoparticles were grown *in situ* onto the Ti_3_C_2_ nanosheet surfaces (Figure 2c). The corresponding elemental mapping (Figure 2d–g) corroborates the coexistence of Ag, N, C, and Ti within TAN-30 and further reveals their homogeneous dispersion throughout the composite.

Transmission electron microscopy (TEM) was employed to further probe the microstructural features of the prepared samples. The ultrasonic exfoliation of Ti_3_C_2_ yielded thin, few-layered nanosheets with lateral dimensions on the order of several micrometers, as clearly observed in Figure 2h. For the TAN-30 composite (Figure 2i), Ag/Ag_2_NCN nanoparticles are seen to be anchored *in situ* onto the Ti_3_C_2_ surfaces, forming a close interfacial contact. Such an intimate junction is expected to facilitate the separation and migration of photogenerated charge carriers, thereby contributing to the enhanced photocatalytic performance. High-resolution TEM imaging (Figure 2j) resolved distinct lattice spacings of 0.204 nm and 0.238 nm, which correspond well to the (200) plane of metallic Ag and the (300) plane of Ag_2_NCN, respectively [31,32]. These findings corroborate the coexistence of Ag and Ag_2_NCN within TAN-30, in full agreement with the XRD results.

**Figure 3:**
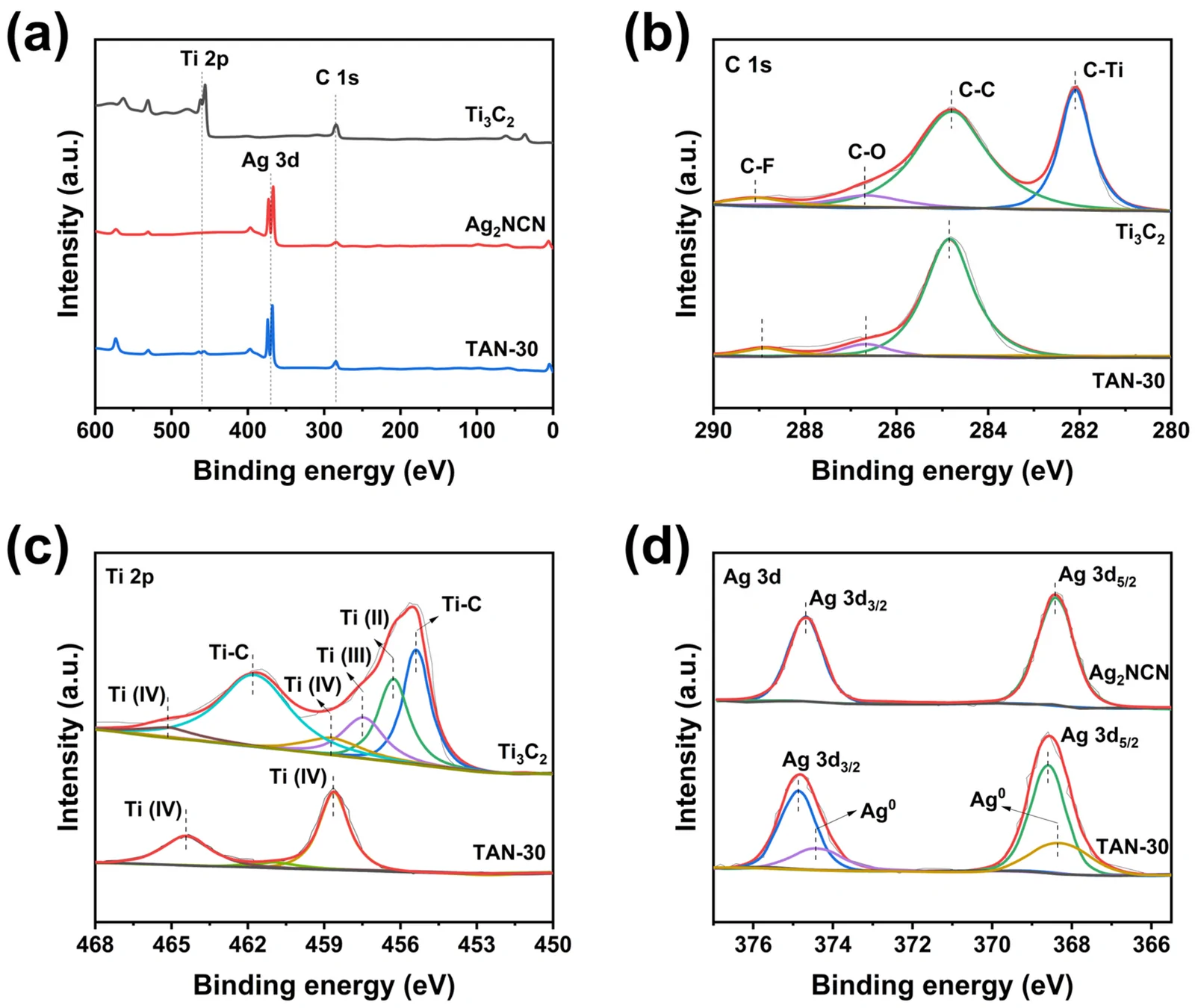
(**a**) Survey spectra of Ti_3_C_2_, Ag_2_NCN, and TAN-30; (**b**) C 1s and (**c**) Ti 2p high-resolution spectra (Ti_3_C_2_ vs. TAN-30); (**d**) Ag 3d high-resolution spectra (Ag_2_NCN vs. TAN-30).

XPS analysis confirmed the elemental composition and interactions in the TAN-30 composite. Figure 3a presents the survey spectra of Ag_2_NCN, Ti_3_C_2_, and TAN-30, and the TAN-30 spectrum shows Ti, C, N, and Ag, consistent with elemental mapping (Figure 2d–g). As shown in Figure 3b, the C 1s spectrum of TAN-30 consists of four peaks at 282.1, 284.8, 286.7, and 289.2 eV, assigned to C–Ti, C–C, C–O, and C–F, respectively [33,34]. The significant attenuation of the C–Ti peak at 282.1 eV suggests that Ag_2_NCN forms a partial surface coating on the Ti_3_C_2_ nanosheets [35]. The Ti 2p spectrum of Ti_3_C_2_ is deconvoluted into six peaks at 455.5, 456.4, 457.3, 458.6, 461.8, and 465.2 eV, corresponding to Ti–C 2p_3/2_, Ti(II), Ti(III), Ti(IV) 2p_3/2_, Ti–C 2p_1/2_, and Ti(IV) 2p_1/2_ (Figure 3c) [36]. The low-valence Ti peaks are absent in TAN-30, implying that low-valence Ti species on the Ti_3_C_2_ surface provide localized electron-rich anchoring sites. These sites reduce the free energy barrier for Ag nucleation via heterogeneous nucleation pathways [37]. Notably, the Ti(IV) binding energies in TAN-30 shift negatively, whereas the Ag 3d peaks shift positively (Figure 3d), revealing electron transfer from Ag_2_NCN to Ti_3_C_2_ that favors charge separation and enhances photocatalytic activity. The Ag 3d spectrum of TAN-30 is resolved into peaks for Ag^+^ (367.8, 373.8 eV) and Ag^0^ (368.1, 374.6 eV), confirming the presence of metallic Ag [38]. To quantify the Ag^0^ and Ag^+^ proportions in the TAN composites, the Ag 3d high-resolution XPS spectra of the TAN samples (TAN-10, TAN-20, TAN-30, and TAN-50) were deconvoluted. The derived peak areas and Ag^0^/Ag^+^ ratios are summarized in Table S1.

### 2.2. Photoelectrochemical Performance

**Figure 4:**
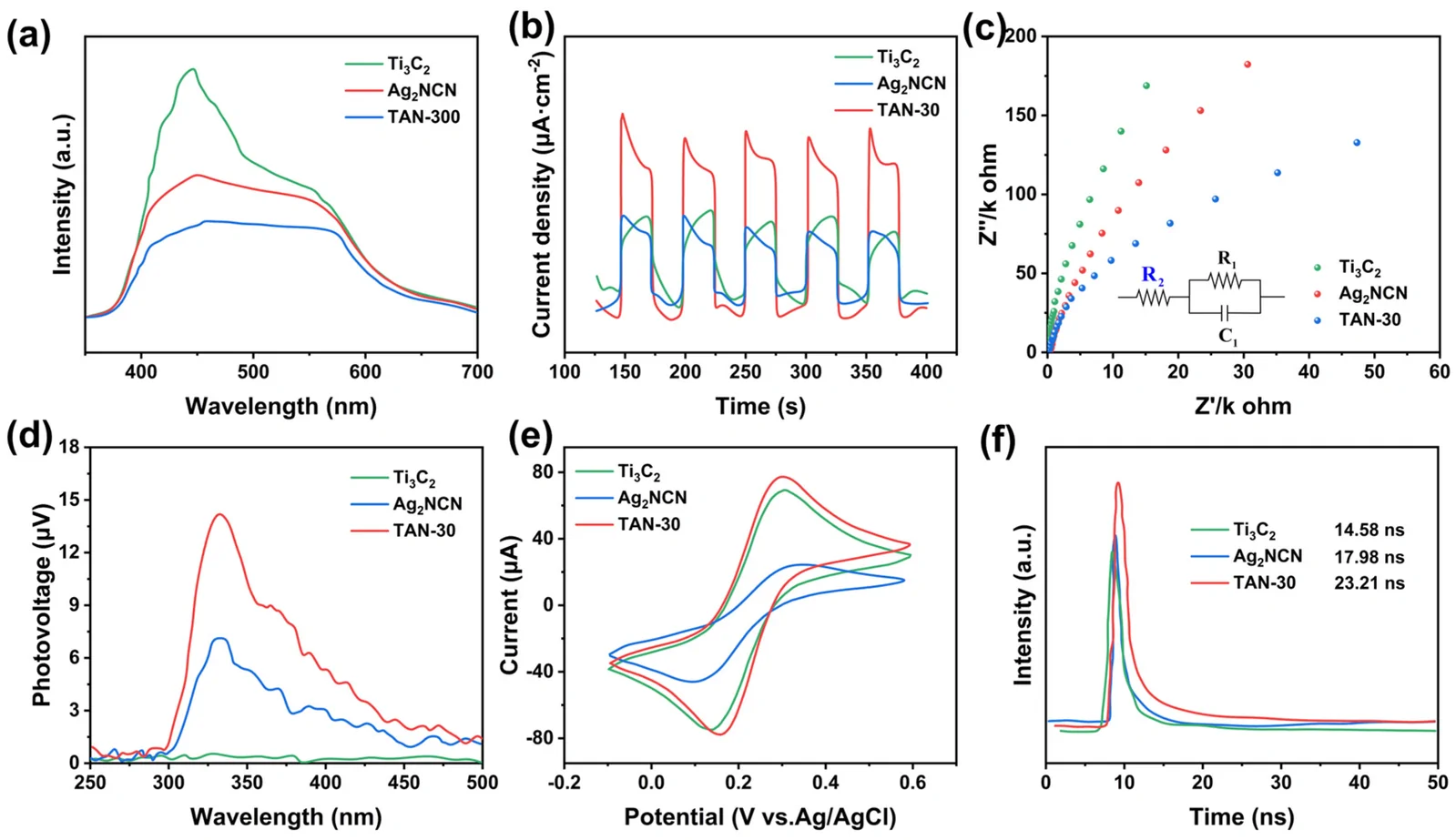
(**a**) PL emission, (**b**) photocurrent density, (**c**) Nyquist plots from EIS, (**d**) SPV intensity, (**e**) CV traces, and (**f**) TR-PL attenuation profiles recorded for Ti_3_C_2_, Ag_2_NCN, and TAN-30 composite.

The efficiency of photogenerated charge separation and migration is pivotal for superior photocatalysis. To probe this aspect of the TAN-30 Schottky catalyst, we combined photoelectrochemical tests with steady-state and transient spectroscopy. Steady-state PL spectra (Figure 4a) revealed that the luminescence intensity of TAN-30 was lower than that of pristine Ti_3_C_2_ and Ag_2_NCN, indicating that their integration suppresses carrier recombination and promotes interfacial charge transfer, thus benefiting the photocatalytic reaction [39]. Transient photocurrent response (TPR) and electrochemical impedance spectroscopy (EIS) were further applied to the three samples to track charge transport dynamics. As shown in Figure 4b, TAN-30 exhibited a markedly higher photocurrent than the individual components during repeated on–off cycles, confirming its enhanced carrier separation capability [40]. Electrochemical impedance spectroscopy (EIS) was employed to probe the interfacial charge transfer kinetics (Figure 4c), where a diminished Nyquist arc radius signifies a lower charge transfer resistance [41]. The heterojunction exhibited a notably smaller arc diameter than either Ti_3_C_2_ or Ag_2_NCN, confirming that interfacing these two components effectively lowers the energy barrier for interfacial charge transfer and suppresses electron–hole pair recombination.

In line with this observation, the TAN-30 heterojunction delivered a substantially intensified surface photovoltage (SPV) response (Figure 4d). Both the SPV and photocurrent signals exhibited positive polarity, a feature characteristic of n-type semiconductors, where photogenerated holes tend to accumulate at the surface. Cyclic voltammetry (CV) measurements were further performed to evaluate the redox capability of the samples (Figure 4e). Compared with the pristine counterparts, the CV curve of TAN-30 presented a larger enclosed peak area, implying that the synergistic coupling between Ti_3_C_2_ and Ag_2_NCN is conducive to the oxidative degradation pathway [16]. To gain further insight into the carrier transport behavior, steady-state and time-resolved photoluminescence (PL) spectroscopy were carried out under 420 nm excitation. Upon the introduction of TC into the system, a marked improvement in carrier migration efficiency was observed. Specifically, TAN-30 showed pronounced PL quenching, while its transient carrier lifetime reached 23.21 ns—substantially longer than those of Ag_2_NCN (14.58 ns) and Ti_3_C_2_ (17.98 ns), further underscoring the beneficial role of heterojunction construction in prolonging charge separation [42]. These findings point to a synergistic mechanism in which the Schottky barrier and the localized surface plasmon resonance (LSPR) of Ag nanoparticles jointly facilitate photogenerated carrier separation and transport within TAN 30, accounting for its markedly enhanced photocatalytic activity.

**Figure 5:**
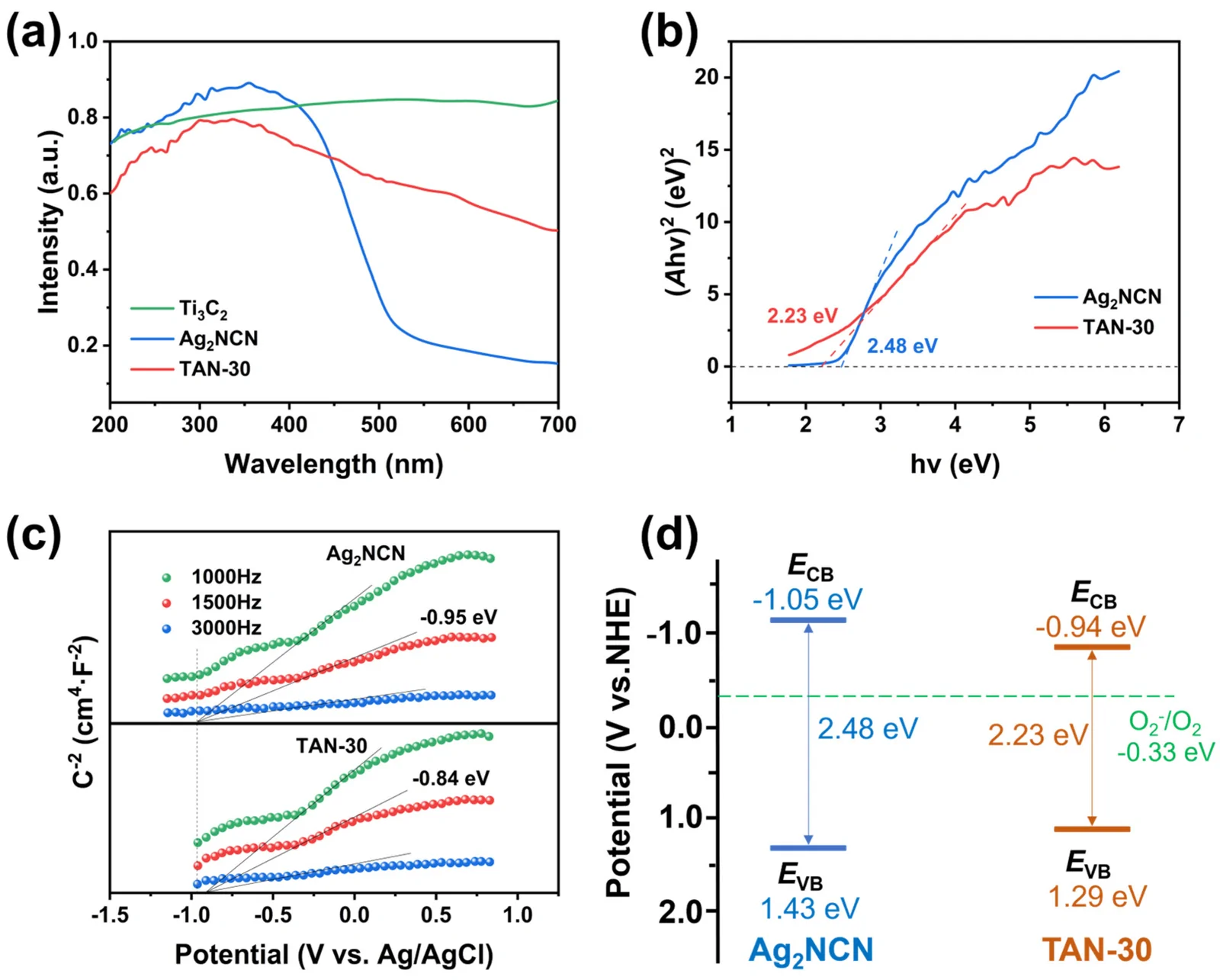
(**a**) UV–Vis DRS of Ti_3_C_2_, Ag_2_NCN, and TAN-30 composite. (**b**) Tauc plots of Ti_3_C_2_, Ag_2_NCN, and TAN-30 composite. (**c**) Mott–Schottky of Ti_3_C_2_, Ag_2_NCN, and TAN-30 composite. (**d**) Energy band diagram of samples.

The band structure of the photocatalysts was probed by UV–visible diffuse reflectance spectroscopy (UV-DRS). As shown in Figure 5a, Ag_2_NCN exhibits an optical absorption edge at around 510 nm. In contrast, the TAN-30 heterojunction displays a clear red shift relative to pristine Ag_2_NCN. Moreover, a pronounced enhancement in light absorption is observed for TAN-30 over the 450–700 nm range, which is chiefly attributable to the surface plasmonic effect arising from Ag clusters [43]. Band gap energies were obtained by fitting the absorption data to the Tauc equation, (αhv) ^1/m^ = *A*·(hv − Eg) [44], in which α, hv, and A denote the absorption coefficient, photon energy, and a constant, respectively. To determine the exponent m, we referred to the Mott–Schottky analysis. The positive slopes in Figure 5c for both Ag_2_NCN and TAN-30 confirm their n-type character, which implies an indirect transition (m = 2). The linear extrapolation of the Tauc plots (Figure 5b) then provided band gaps of 2.48 eV and 2.23 eV for Ag_2_NCN and TAN-30, respectively. Mott–Schottky measurements were further conducted to determine the band edge positions of the photocatalysts. As shown in Figure 5c, the flat-band potentials (E_fb_) of Ag_2_NCN and TAN-30 were found to be −0.95 and −0.84 V vs. Ag/AgCl, respectively. The conduction band (E_CB_) is typically taken as ca. 0.1 eV more negative than Efb for n-type semiconductors. Accordingly, the E_CB_ values of Ag_2_NCN and TAN-30 were estimated to be −1.05 and −0.94 eV, respectively. Using the relation E_VB_ = E_CB_ + E_g_, the corresponding valence band potentials (E_VB_) were calculated as 1.43 and 1.29 eV [38]. The resulting band alignment is schematically illustrated in Figure 5d. Notably, the redox potential of O_2_/•O_2_^−^ (−0.33 eV) falls within the band gap, suggesting that the •O_2_^−^ species may participate in the degradation process.

### 2.3. Photocatalytic Performance

**Figure 6:**
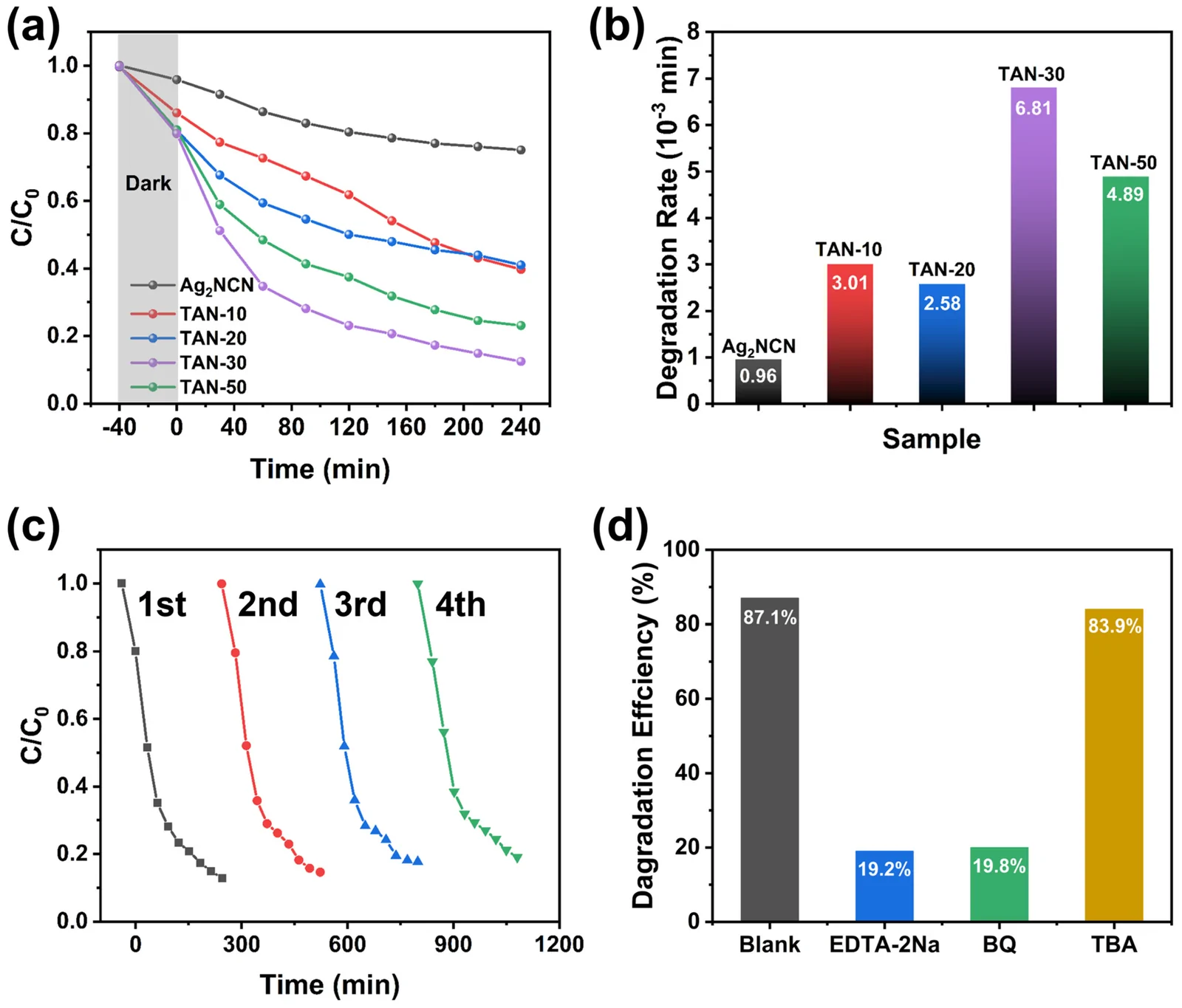
(**a**) Photocatalytic degradation performance of tetracycline over as-prepared samples; (**b**) corresponding pseudo-first-order kinetic linear fittings. (**c**) Reusability test of TAN-30 through cyclic runs; (**d**) radical trapping experiments for identifying main active species in TAN-30 system.

The photocatalytic degradation of tetracycline over Ag_2_NCN and Ti_3_C_2_/Ag–Ag_2_NCN (TAN) composites was evaluated under visible light irradiation (400–800 nm). As presented in Figure 6a, all TAN samples exhibited enhanced adsorption capacity during the dark equilibration stage relative to pristine Ag_2_NCN. Bare Ag_2_NCN showed negligible photoactivity, removing only 24.6% of tetracycline after 240 min of illumination. In contrast, the integration of Ti_3_C_2_ substantially boosted degradation efficiency, with the optimal TAN-30 composition achieving the highest removal rate of approximately 87.0% under identical conditions. The degradation kinetics were well described by a pseudo-first-order model (Figure 6b). The decolorization and structural destruction of TC do not necessarily equate to complete mineralization, as the generation of smaller organic intermediates may still pose environmental concerns. To evaluate the mineralization degree, total organic carbon (TOC) and chemical oxygen demand (COD) were monitored during the photocatalytic degradation of RhB over TAN-30. After 240 min of visible light irradiation, the removal efficiencies reached 59.2% for TOC and 66.8% for COD (Figure S4). Both values were lower than the corresponding TC degradation efficiency, which is attributable to the progressive accumulation of organic intermediates throughout the photocatalytic process. Nevertheless, the TOC data confirm that over 59.2% of the initial organic carbon in the RhB solution was ultimately mineralized to CO_2_. The rate constant of TAN-30 was determined to be 6.81 × 10^−3^ min^−1^, which is roughly 7.0 times that of pure Ag_2_NCN. To exclude the contributions of physical adsorption and increased specific surface area, a control experiment using a physically Ti_3_C_2_/Ag_2_NCN mix sample was performed (Figure S1). The marginal enhancement observed in the physical mixture, compared to the chemically bonded composite, confirms that the dominant mechanism is interfacial charge transfer (Schottky effect) and plasmonic resonance coupling, rather than mere textural or adsorptive effects. Furthermore, recycling experiments confirmed the satisfactory stability of TAN-30 over repeated degradation cycles.

The reusability and structural stability of TAN-30 were assessed through cyclic photocatalytic experiments (Figure 6c). Over four successive runs, no appreciable loss in tetracycline (TC) degradation activity was observed, confirming the satisfactory recyclability of the composite. The structural stability of the TAN-30 composite was further investigated by comparing the XRD patterns of the fresh and used catalysts after the cycling tests (Figure S3). As shown in Figure S3, no discernible changes in peak positions or intensities were observed for either the Ti_3_C_2_ or metallic Ag components, confirming the absence of photocorrosion or oxidation during the photocatalytic reaction. To identify the primary reactive species involved in the degradation process, radical trapping experiments were carried out using ethylenediaminetetraacetic acid disodium salt (EDTA-2Na), benzoquinone (BQ) and tert-butanol (TBA) as quenchers for holes (h^+^), superoxide radicals (•O_2_^−^) and hydroxyl radicals (•OH), respectively [45]. As displayed in Figure 6d, the photocatalytic degradation efficiency in the absence of any scavenger (blank) reached 87.1%. Upon the addition of EDTA-2Na (1.0 mM), a hole (h^+^) scavenger, and p-benzoquinone (BQ, 1.0 mM), a superoxide radical (•O_2_^−^) scavenger, the degradation efficiencies were dramatically suppressed to 19.2% and 19.8%, respectively. This significant inhibition indicates that h^+^ and •O_2_^−^ are the two dominant reactive species governing the photocatalytic reaction. In contrast, the addition of tert-butanol (TBA, 1.0 mM), a hydroxyl radical (•OH) scavenger, resulted in only a marginal decrease from 87.1% to 83.9%. This minor suppression suggests that •OH plays only a negligible role in the degradation process.

**Figure 7:**
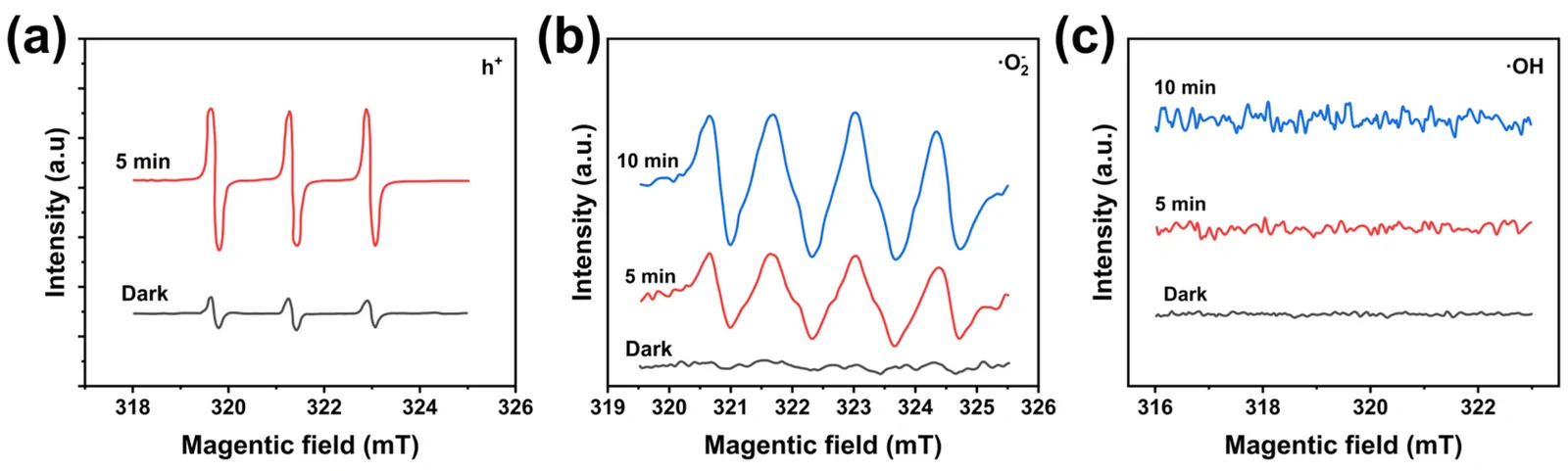
ESR spectra of TEMPO-h^+^, DMPO-•O_2_^−^, and DMPO-•OH adducts in TAN-30 dispersion under Xe lamp irradiation: (**a**) h^+^, (**b**) •O_2_^−^, (**c**) •OH.

Spin-trapping ESR measurements were performed to identify the reactive species involved in the photocatalytic degradation of tetracycline. DMPO and TEMPO were used as spin traps for •O_2_^−^, •OH and h^+^, respectively [46]. As shown in Figure 6, no characteristic DMPO-•O_2_^−^ or DMPO-•OH signals were detected in the dark, while a weak TEMPO-h^+^ peak was observed. Upon irradiation with a 300 W Xe lamp, the TEMPO-h^+^ signal increased markedly, and the DMPO–•O_2_^−^ peaks grew progressively with extended illumination (Figure 7a,b). In contrast, no clear •OH-derived peaks were resolved, although a slight rise in the baseline signal was noted (Figure 7c). These observations confirm that both superoxide radicals and photogenerated holes participate in the photocatalytic process.

### 2.4. Analysis of Photocatalytic Mechanism

**Figure 8:**
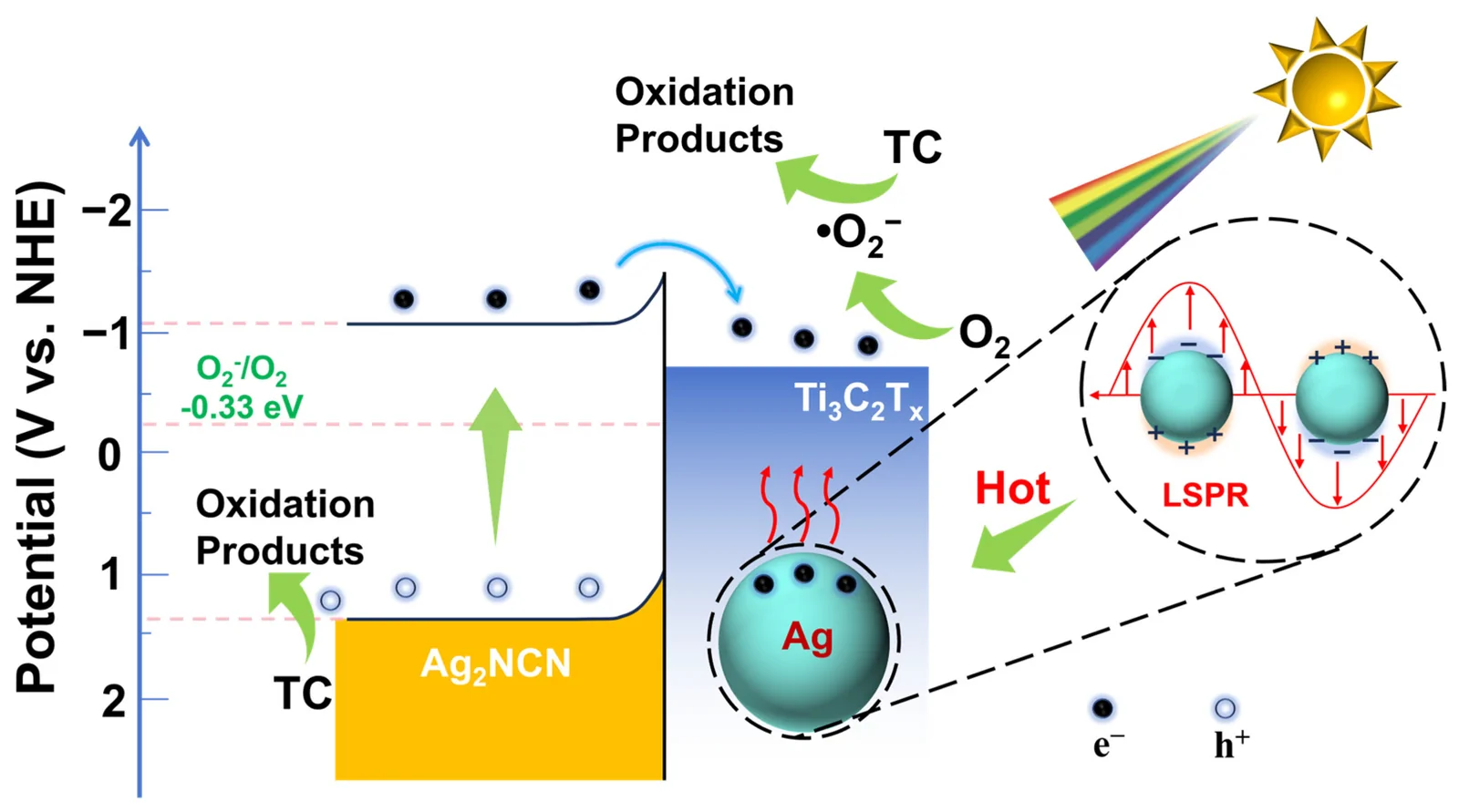
A schematic illustration of the charge transfer and ROS generation mechanism over the TAN Schottky heterojunction.

The photothermal response of the photocatalysts was evaluated by tracking the sample temperature via thermal imaging throughout visible light irradiation (Figure S2). A temperature gap of approximately 20 °C was observed between the TAN-30 composite and the neat Ag_2_NCN at the end of the illumination period, with the former reaching a markedly higher steady-state temperature. Although this photothermal conversion capability is notable, the photocatalytic experiments were conducted at a controlled temperature of 6 °C, where macroscopic photothermal heating is effectively suppressed. Therefore, the LSPR effect of Ag^0^ is proposed to operate predominantly via a nonthermal mechanism, namely the injection of hot electrons from plasmon-excited Ag^0^ into the adjacent Ag_2_NCN and Ti_3_C_2_ components. The proposed mechanism for the enhanced reactive oxygen species (ROS) generation over the TAN Schottky heterojunction under visible–near-infrared (vis–NIR) irradiation is illustrated in Figure 8. Upon photoexcitation, electrons in Ag_2_NCN are promoted to the conduction band and migrate across the Schottky interface to Ti_3_C_2_. The built-in Schottky barrier effectively suppresses charge recombination, thus promoting electron–hole separation. Concurrently, hot electrons generated from the LSPR-excited Ag^0^ are injected into the conduction band of Ag_2_NCN or directly into Ti_3_C_2_, further contributing to charge density. Meanwhile, the high electrical conductivity of layered Ti_3_C_2_ facilitates rapid electron transport, synergistically enhancing carrier separation. As a result, the optimal integration of Ag–Ag_2_NCN with Ti_3_C_2_ leads to significantly enhanced ROS production.

## 3. Materials and Methods

### 3.1. Chemicals and Reagents

Ti_3_AlC_2_ powder (purity ≥98%, 200 mesh) was purchased from Jilin 11 Technology Co., Ltd. (China). Sodium fluoride (NaF, ≥99.0%, AR), hydrochloric acid (HCl, 36.0–38.0%, GR), ammonia solution (NH_3_·H_2_O, 25.0–28.0%, AR), and absolute ethanol (≥99.7%, AR) were obtained from Sinopharm Chemical Reagent Co., Ltd. (China). Silver nitrate (AgNO_3_, ≥99.8%, AR) and sodium borohydride (NaBH_4_, ≥98.0%, AR) were purchased from Shanghai Aladdin Biochemical Technology Co., Ltd. (China). Cyanamide (H_2_NCN, ≥98.0%, AR) was supplied by Shanghai Macklin Biochemical Co., Ltd. (China). Deionized (DI) water (resistivity ≥18.2 MΩ·cm) was produced by a Millipore Direct-Q UV water purification system and used throughout all experiments. All chemicals were used as received without further purification.

### 3.2. Synthesis of Ti_3_C_2_T_x_

Ti_3_C_2_T_x_ was synthesized via the selective etching of Ti_3_AlC_2_ using a solution prepared by dissolving NaF (5.0 g) in 40.0 mL of hydrochloric acid (9.0 M) [47]. Typically, 2.0 g of Ti_3_AlC_2_ powder was gradually added to the etching solution (40.0 mL), and the suspension was stirred at 35 °C for 72 h. The resulting product was washed repeatedly with HCl and deionized water until the pH exceeded 6, then collected by centrifugation (8000 rpm, 5 min) and dried under vacuum at 60 °C overnight.

### 3.3. Synthesis of Ag_2_NCN

AgNO_3_ (0.85 g) was dissolved in deionized water (20 mL), followed by the rapid addition of NH_3_·H_2_O solution (1.5 M, 125.0 mL) under magnetic stirring to form a clear solution. The dropwise addition of cyanamide (H_2_NCN) aqueous solution (5.0 mL, containing 0.21 g, 5.0 mmol) caused the mixture to turn golden yellow with immediate precipitation. The precipitate was collected by centrifugation (3500 rpm, 5 min), washed three times with deionized water, and dried under vacuum at 60 °C overnight [22].

### 3.4. Synthesis of Preparation of Ti_3_C_2_/Ag–Ag_2_NCN Composites

A series of Ti_3_C_2_/Ag–Ag_2_NCN composites with varied Ag loadings, denoted as TAN-x (x = 10, 20, 30, 50), was prepared via an *in situ* precipitation–chemical reduction route. In a typical procedure, Ti_3_C_2_T_x_ powder (0.05 g) was dispersed in deionized water (20 mL) by ultrasonication for 30 min. A specified amount of AgNO_3_ (10, 20, 30, and 50 mmol) was dissolved in the above dispersion, followed by the rapid addition of NH_3_·H_2_O solution (1.5 M, 125.0 mL) under magnetic stirring to form a clear solution. The dropwise addition of an aqueous cyanamide (H_2_NCN) solution (with the same molar amount as AgNO_3_, in a total volume of 5.0 mL) immediately generated a golden-yellow precipitate of Ag_2_NCN, which deposited onto the Ti_3_C_2_ sheets. After stirring for 30 min, an aqueous NaBH_4_ solution was added dropwise over 10 min under vigorous stirring, reducing the Ag^+^ to metallic Ag^0^. The suspension was stirred for an additional 1 h. The final solid was collected by centrifugation, washed repeatedly with deionized water, and dried under vacuum at 60 °C overnight.

### 3.5. Photocatalytic Measurement

The photocatalytic degradation of tetracycline (TC) was carried out in a quartz photoreactor under a 300 W Xe lamp (PLS-SXE300, Beijing Perfectlight Technology Co., Ltd., China) equipped with a UV cutoff filter (λ > 420 nm) used as the visible light source. The spectrum confirms that the lamp predominantly emits in the visible light region (420–800 nm) with negligible UV components below 420 nm. The distance between the lamp and the surface of the reaction solution was fixed at 15 cm. In a typical experiment, 20 mg of the catalyst was dispersed in 100 mL of TC aqueous solution (20 mg·L^−1^). Before illumination, the suspension was magnetically stirred in the dark for 40 min to establish adsorption–desorption equilibrium. The reaction temperature was maintained at 6 °C by a circulating water jacket. At regular intervals, aliquots of the suspension were sampled, filtered through a 0.22 μm membrane, and analyzed using a UV-Vis spectrophotometer to monitor the residual TC concentration. Degradation efficiency was calculated from the change in absorbance at the characteristic absorption peak of TC. The concentration of tetracycline (TC) during the photocatalytic degradation experiments was determined by measuring absorbance at its characteristic absorption wavelength (λmax = 357 nm) using a UV-Vis spectrophotometer (Shimadzu UV-2600).

### 3.6. Reusability and Radical Trapping Experiments

To evaluate the stability and reusability of the TAN-30 composite, the photocatalyst was recovered after each photocatalytic degradation run by centrifugation at 8000 rpm for 10 min. The recovered catalyst was subsequently washed three times with deionized water and once with absolute ethanol, then dried in an oven at 60 °C for 12 h. The dried catalyst was directly reused in the next cycle under identical reaction conditions without any further regeneration. This recycling procedure was repeated for 4 consecutive runs, and the degradation efficiency of each cycle was recorded.

For the radical trapping experiments, the photocatalytic degradation of TC was carried out under identical conditions in the presence of various radical scavengers to identify the main reactive oxygen species (ROS). Specifically, tert-butanol (TBA, 1.0 mM) was employed as a hydroxyl radical (•OH) scavenger, benzoquinone (BQ, 1.0 mM) as a superoxide radical (•O_2_^−^) scavenger, and ethylenediaminetetraacetic acid disodium salt (EDTA-2Na, 1.0 mM) as a photogenerated hole (h^+^) scavenger. All scavengers were added to the TC solution prior to the addition of the photocatalyst and light irradiation. The photocatalytic activity in the presence of each scavenger was compared to that of the control experiment (without scavenger) performed under the same conditions.

### 3.7. Sample Characterizations

Powder X-ray diffraction (XRD) patterns were collected on a Bruker D8 Advance diffractometer with Cu Kα radiation (λ = 1.5418 Å) over 2θ = 5–80°. Raman spectra were recorded on a Renishaw inVia confocal microscope using 532 nm laser excitation. Scanning electron microscopy (SEM) and energy-dispersive X-ray spectroscopy (EDS) mapping were performed on a Zeiss Sigma 300 field-emission SEM, while transmission electron microscopy (TEM) and high-resolution TEM (HRTEM) were conducted on an FEI Talos F200X operated at 200 kV. X-ray photoelectron spectroscopy (XPS) was carried out on a Thermo Scientific K-Alpha spectrometer with monochromatic Al Kα radiation, and binding energies were calibrated using the C 1s peak at 284.8 eV. Nitrogen adsorption–desorption isotherms were measured at 77 K on a Micromeritics ASAP 2460 analyzer, with specific surface areas determined by the Brunauer–Emmett–Teller (BET) method. UV–vis diffuse reflectance spectra (UV–Vis DRS) were obtained on a Shimadzu UV-2600 spectrophotometer fitted with an integrating sphere, using BaSO_4_ as a reference. Photoluminescence (PL) and time-resolved PL (TRPL) decay profiles were acquired on an Edinburgh Instruments FLS1000 spectrometer at an excitation wavelength of 420 nm. Electrochemical measurements—including transient photocurrent response, electrochemical impedance spectroscopy (EIS), Mott–Schottky analysis, and cyclic voltammetry (CV)—were performed on a CHI 760E workstation in a standard three-electrode cell with a Pt wire counter electrode, an Ag/AgCl reference electrode, and a catalyst-coated FTO working electrode in 0.5 M Na_2_SO_4_ aqueous solution. Surface photovoltage (SPV) measurements were conducted on a home-built system with a lock-in amplifier. Electron spin resonance (ESR) spectra were recorded on a Bruker E500 spectrometer using DMPO and TEMPO as spin traps for •O_2_^−^/•OH and h^+^, respectively, under both dark and light irradiation.

## 4. Conclusions

In summary, a series of Ti_3_C_2_/Ag-Ag_2_NCN (TAN) Schottky heterojunctions with varying Ag loadings was successfully fabricated via an *in situ* precipitation–chemical reduction route, during which metallic Ag^0^ was generated concurrently with Ag_2_NCN deposition onto Ti_3_C_2_T_x_ nanosheets. Systematic characterizations confirmed the formation of intimate interfacial contacts among the three components, with Ag^0^ serving dual roles as a plasmonic photosensitizer and an electron mediator. The optimized TAN-30 composite exhibited a tetracycline degradation efficiency of 87.0% under visible light irradiation within 240 min, with a pseudo-first-order rate constant approximately 7.0 times that of pristine Ag_2_NCN. The enhanced photocatalytic performance is attributed to the synergistic interplay between the Schottky barrier at the Ti_3_C_2_/Ag_2_NCN interface and the LSPR effect of *in situ* formed Ag^0^. The Schottky junction facilitates unidirectional electron transfer from Ag_2_NCN to Ti_3_C_2_, effectively suppressing charge recombination, while the LSPR effect broadens the light absorption range and promotes hot electron injection. This work not only demonstrates the rational design of MXene-based plasmonic heterojunctions through *in situ* reduction strategies but also provides mechanistic insights into the cooperative enhancement in charge separation and light harvesting.

## Data Availability

The authors confirm that the data supporting the findings of this study are available within the article.

## Supplementary Materials

The following supporting information can be downloaded at https://www.mdpi.com/article/10.3390/molecules31172955/s1, Figure S1: Photocatalytic degradation performance of tetracycline over the Ti_3_C_2_/Ag_2_NCN-mix samples; Figure S2: Temperature change of Ag_2_NCN and TAN-30 under/after VL (inset: IR images); Figure S3: XRD patterns of the TAN-30 after cycling four times; Figure S4: The COD and TOC removal rate of TC over TAN-30 nanocomposite; Table S1: Peak area and peak area ratio of Ag^+^ and Ag^0^ in TAN composites from Ag 3d high-resolution XPS.
