# Supplementary material for: In Situ Reduction-Generated Ag0 Plasmonic Sites on Ti3C2/Ag2NCN Schottky Heterojunctions for Efficient Photocatalytic Tetracycline Degradation
Source: Molecules. 2026 Aug 23;31(17):2955. doi: 10.3390/molecules31172955 (PMC13566968; doi:10.3390/molecules31172955)
Supplement: Supplementary file 1 [file molecules-31-02955-s001.zip › molecules-4496481-supplementary.pdf]

Supplementary Information for

In Situ Reduction-Generated Ag^0 Plasmonic Sites on Ti_3C_2/Ag_2NCN Schottky Heterojunctions for Efficient Photocatalytic Tetracycline Degradation

Haidong Yu [1], Hua Deng [1], Jincheng Wang [1], Xiaohe Sun [1], Jingyu Liu [1], Ping Qu [2,*] and Jie Wu [1,*]

[1] Langfang Natural Resources Comprehensive Survey Center, China Geological Survey, Langfang 065000, China; yuhaidong1992@163.com (H.Y.); denghua137@126.com (H.D.); 18129361330@163.com (J.W.); 18953588146@163.com (X.S.); 17692600460@163.com (J.L.)

[2] Center for Geophysical Survey, China Geological Survey, Langfang 065000, China

* Correspondence: quping_cgs@163.com (P.Q.); wujie100123@163.com (J.W.)

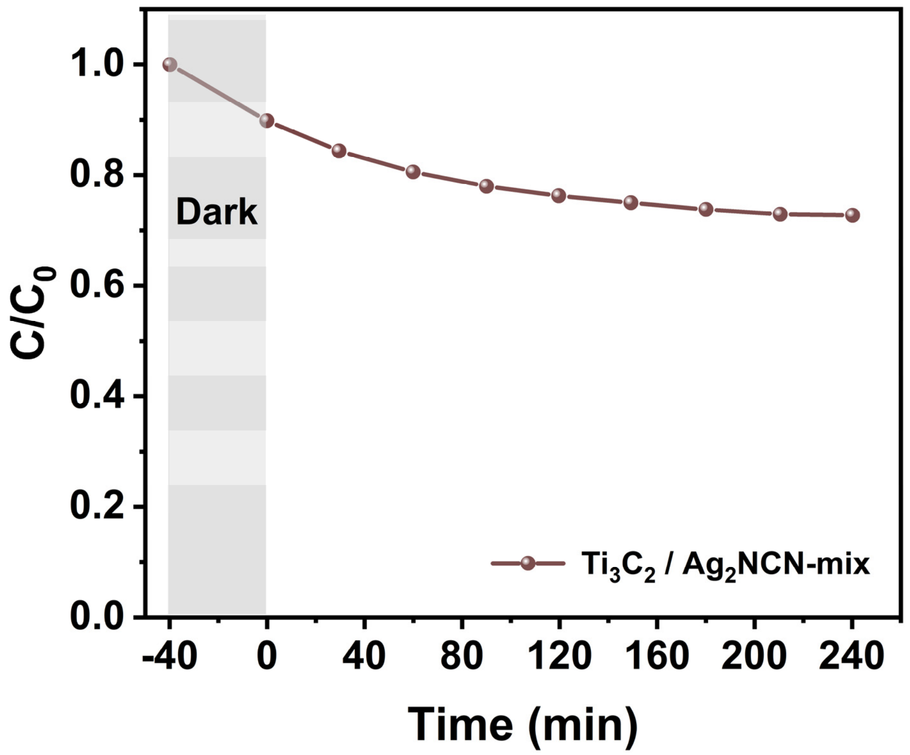

Figure S1. Photocatalytic degradation performance of tetracycline over the Ti_3C_2/Ag_2NCN-mix samples.

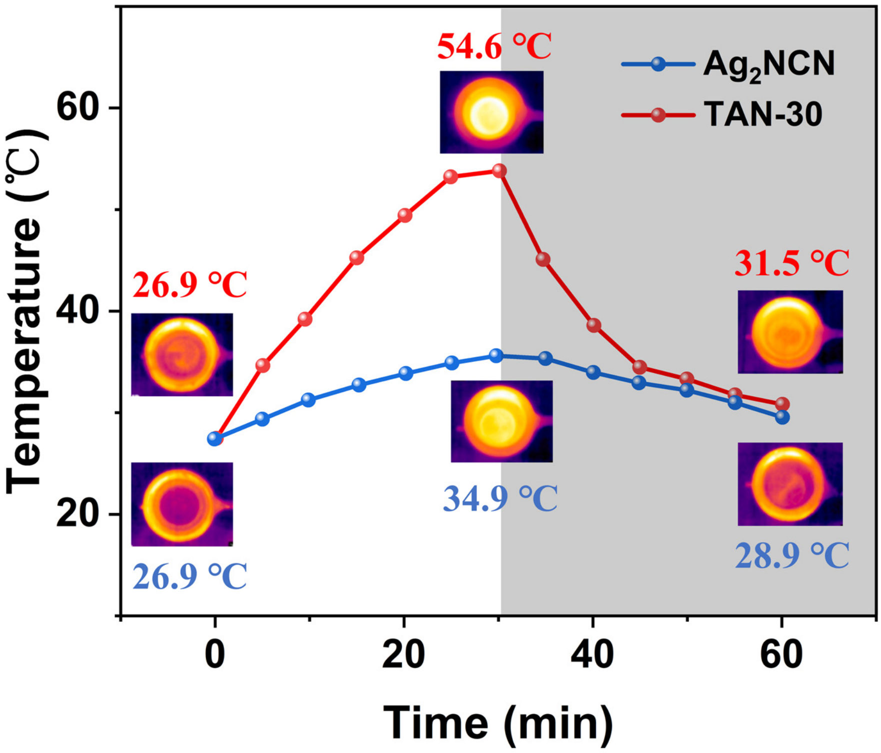

Figure S2. Temperature change of Ag_2NCN and TAN-30 under/after VL (inset: IR images).

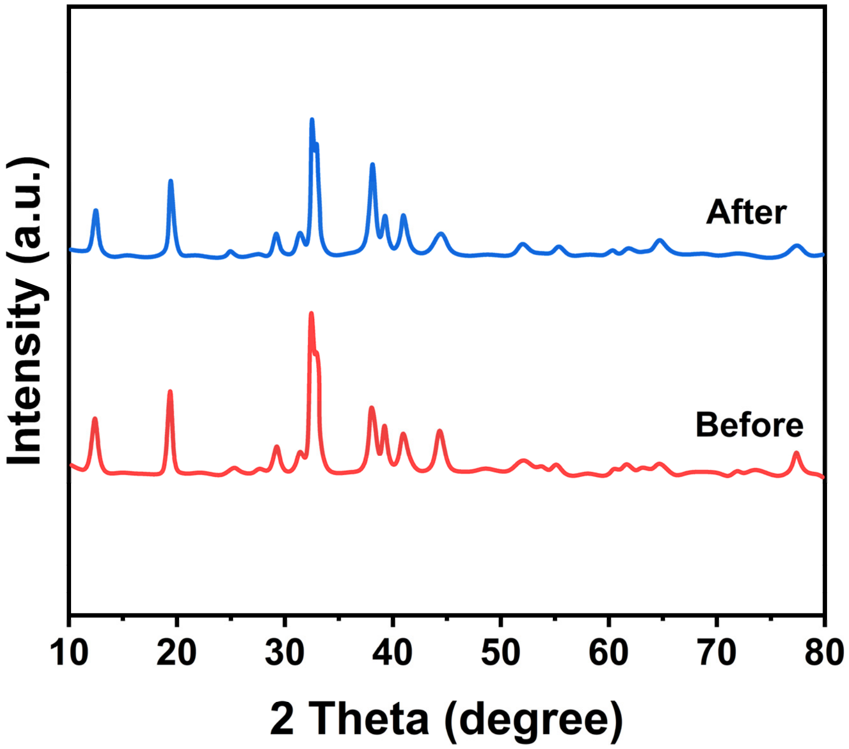

Figure S3. XRD patterns of the TAN-30 after cycling four times.

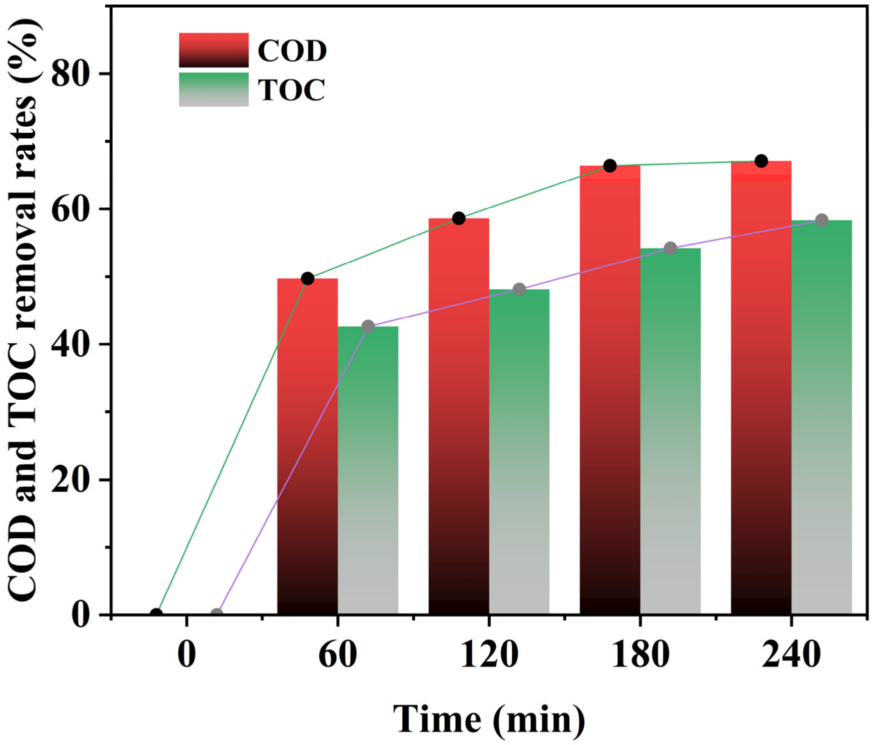

Figure S4. The COD and TOC removal rate of TC over TAN-30 nanocomposite.

Table S1. Peak area and peak area ratio of Ag^{+} and Ag^{0} in TAN composites from Ag 3d high-resolution XPS.

Samples	**Ag^{0} peak area**	**Ag^{+} peak area**	**Ag^{0}/ Ag^{+} peak area ratio**
**TAN-10**	7538.2	23184.6	1:3.08
**TAN-20**	8416.8	25126.9	1:2.99
**TAN-30**	9517.1	27435.2	1:2.88
**TAN-50**	10725.9	28985.3	1:2.71
